# Subregional Hippocampal Thickness Abnormalities in Older Adults with a History of Heavy Cannabis Use

**DOI:** 10.1089/can.2018.0035

**Published:** 2018-12-10

**Authors:** Alison C. Burggren, Prabha Siddarth, Zanjbeel Mahmood, Edythe D. London, Theresa M. Harrison, David A. Merrill, Gary W. Small, Susan Y. Bookheimer

**Affiliations:** ^1^ Semel Institute for Neuroscience and Human Behavior, University of California, Los Angeles, California.; ^2^ Center for Cognitive Neurosciences, University of California, Los Angeles, California.; ^3^ San Diego Joint Doctoral Program in Clinical Psychology, San Diego State University/University of California, San Diego, California.; ^4^ Department of Molecular and Medical Pharmacology, University of California, Los Angeles, California.; ^5^ Helen Wills Neuroscience Institute, University of California, Berkeley, California.

**Keywords:** hippocampus, magnetic resonance imaging, high-resolution, cortical thickness

## Abstract

**Background and Aims:** Legalization of cannabis (CB) for both medicinal and, in some states, recreational use, has given rise to increasing usage rates across the country. Of particular concern are indications that frequent CB use may be selectively harmful to the developing adolescent brain compared with adult-onset usage. However, the long-term effects of heavy, adolescent CB use on brain structure and cognitive performance in late-life remain unknown. A critical brain region is the hippocampus (HC), where there is a striking intersection between high concentrations of cannabinoid 1 (CB1) receptors and age-related pathology.

**Design:** We investigated whether older adults (average age=66.6+7.2 years old) with a history of early life CB use show morphological differences in hippocampal subregions compared with older, nonusers.

**Methods:** We performed high-resolution magnetic resonance imaging combined with computational techniques to assess cortical thickness of the medial temporal lobe, neuropsychological testing, and extensive drug use histories on 50 subjects (24 formerly heavy cannabis users [CB+ group] abstinent for an average of 28.7 years, 26 nonusers [CB− group]). We investigated group differences in hippocampal subregions, controlling for age, sex, and intelligence (as measured by the Wechsler Test of Adult Reading), years of education, and cigarette use.

**Results:** The CB+ subjects exhibited thinner cortices in subfields cornu ammonis 1 [CA1; F(1,42)=9.96, *p*=0.0003], and CA2, 3, and the dentate gyrus [CA23DG; F(1,42)=23.17, *p*<0.0001], and in the entire HC averaged over all subregions [F(1,42)=8.49, *p*=0.006].

**Conclusions:** Negative effects of chronic adolescent CB use on hippocampal structure are maintained well into late life. Because hippocampal cortical loss underlies and exacerbates age-related cognitive decline, these findings have profound implications for aging adults with a history of early life usage.

Clinical Trial Registration: ClinicalTrials.gov # NCT01874886.

## Introduction

Marijuana (*Cannabis sativa*) is the most widely used “illicit” drug worldwide, with an estimated 183 million past-year users worldwide,^1^ over 20 million Americans reporting previous month use, and 4.6 million Americans meeting criteria for dependence in 2015.^2^ Adolescent cannabis (CB) use is particularly concerning given the vulnerability of the adolescent brain.^3^ Since brain development continues throughout young adulthood,^4^ adolescence may be a critical period during which CB exposure may have long-lasting implications.^5^ In animal models, the primary psychoactive ingredient in CB, Δ9-tetrahydrocannabinol (THC) induces dose-dependent neurotoxic changes in the brain.^6,7^ Specific brain regions affected, including the hippocampus (HC), amygdala, striatum, and cingulate cortex, have high densities of cannabinoid 1 (CB1) receptors^8^ and, importantly, are among the first brain areas to show age-related morphological changes.^9^

For several decades, results regarding whether chronic CB use damages the brain were mixed.^10–13^ However, technological advances in magnetic resonance imaging (MRI) have enabled findings that *chronic* CB use has a significant effect on hippocampal structure in adolescents,^5,14–17^ and mounting evidence points to cognitive impairment after heavy CB use.^18–21^ People who were adolescents in the 1960s and 70s, when CB use doubled,^22^ are now entering late life, a period of high risk for age-related memory and cognitive deficits.^23–27^ However, how heavy CB use early in life affects brain structure and cognitive performance in late life is unknown.

We focused the present investigation on hippocampal morphology and cognitive performance because the HC exhibits dense concentrations of CB1 receptors^8,28^ and because it is the primary site of age-related changes associated with memory impairment and dementia.^23,24,29^ Prior MRI studies have suggested a particularly adverse effect of CB on the HC during adolescent brain development.^14^ There is also substantial evidence for neuroanatomical abnormalities within the HC in CB users.^30–32^

One study of heavy CB users found smaller volumes of the HC and amygdala, and provided some of the earliest imaging evidence that heavy, long-term CB use is harmful to brain tissue.^33^ In younger subjects (30 years on average), volumes in hippocampal subregions CA1, 2, 3 and the dentate gyrus (DG) were smaller in dependent CB users than in nonusers,^30^ but similar data were not available from older subjects (over the age of 55) to see whether changes to hippocampal structure persisted over time.

In this study, ultra-high-resolution MRI data focusing on the HC were obtained and analyzed using a cortical unfolding technique, enabling analysis of cortical thickness in hippocampal subregions. This technique has been used to reveal subtle brain differences among cognitively intact older adults,^34–36^ to predict decline in cognition,^37^ and to show changes in the CA1 in multiple sclerosis.^38^

Subregional analysis of the HC has been shown in other laboratories,^39^ and ours,^34^ to be more sensitive to subtle morphological differences in hippocampal subregions than volumetric analyses. Additionally, CB1 receptors are not distributed equally across the HC but are most densely concentrated in CA2, CA3, and the DG, followed by CA1 in decreasing order.^40^ Similarly, cortical thinning in aging is region specific, with entorhinal cortex (ERC) and CA1 particularly affected.^41^

As a control to show that brain differences are specific to regions with high CB1 receptor density, we also conducted a region of interest (ROI) analysis of the parietal cortex, a brain region which also exhibits age-related morphological changes in late life, but which has a lower CB1 receptor density than the medial temporal lobe (MTL).^42^ Hippocampal and parietal cortex morphology were therefore compared between former heavy CB users (“CB+” group) and control subjects (“CB−” group). This is the first study that examines older individuals with a history of early life CB use to answer questions about the longitudinal effects of CB use in the aging brain.

## Methods

The study was conducted with the approval of the UCLA IRB; subjects were recruited from the local community through advertisements in local media resources and signed informed consent forms before participating. Subjects were first screened over the phone and those reporting use of cocaine, methamphetamine, ecstasy, heroin, or other illicit substances more than once were excluded. We recruited older subjects (57–75 years old) with “significant” CB exposure during adolescence (defined as CB use on at least 20 days/month, initiating use during adolescence (before age 20) and continuing for at least 1 year with no more than one to two uses/month after age 35).

We enrolled 24 former heavy CB users (“CB+”) and 26 control subjects (“CB−”) who reported never having used cannabis or any other illicit substances. Participants also provided a urine sample for biological verification of abstinence from use of any illicit substances on the day of neuropsychological testing (Instant-View Multi-Drugs of Abuse Urine Test; Alfa Scientific Designs, Inc.). Participants with a history of neurological or psychiatric disorders, engagement in psychological treatment (within the previous 6 months), or current or past diagnoses of psychotic disorders were excluded from the study using DSM-V criteria.^43^

During their visit, participants underwent neuropsychological testing, a clinical interview, a physical and medical examination, and laboratory screening, including tests to rule out medical conditions that could affect cognitive performance (e.g., abnormal thyroid or pituitary hormone levels).^44,45^ Subjects with a history of uncontrolled hypertension or cardiovascular disease (systolic blood pressure [BP] >170 or diastolic BP >100), head trauma, or other major systemic disease affecting brain function were excluded. Participants taking medications that could influence psychometric testing were also excluded.^46^ Drug use characteristics were assessed through a semistructured interview (“Drug Use History;” Table 1), which was used to characterize lifetime and current substance use.

**Table 1.** **Drug Abuse Phenotyping (1 h)**

Measure	Description	Variables	Duration	No. of Items
Fagerstrom Test for Nicotine Dependence^70^	Self-report assessment (six items) of smoking behavior (e.g., time to first cig, cigs/day) and dependence.	Smoking behavior, nicotine dependence	∼3 min Smokers only	12
Cigarette Use Timeline (London Lab, unpublished)	Self-report form. Participant to fill in smoking rates on a timeline of his/her smoking history. Used to estimate pack-years.	Cigarettes/day over the smoking history	5–10 min Smokers only	N/A
Drug Use History (London Lab, unpublished)	Structured interview utilizing a standard form. Collects data regarding use of drugs of abuse in all classes (age at first use, current usage, greatest regular lifetime usage, and peak usage).	Complete drug use history	15 min	1011
Marijuana Dependence Checklist^71^	Self-report questionnaire. Items assess dependence on cannabis.	Cannabis dependence	2–3 min MJ smoker only	10
Marijuana Smoking History Questionnaire^52^	Self-report questionnaire. Collects data regarding recent and lifetime cannabis use, average amounts of use and routes of administration. Also asks about changes in use, and quit attempts.	Cannabis use	1–8 min	22
Smoking History Questionnaire (London Lab, unpublished)	Self-report form. Items relate to benchmarks of smoking behavior (e.g., age at first cigarette, age at first 100 cigarettes, longest quit attempt, number of quit attempts, reasons for quitting)	Data related to smoking behavior	5 min	26
Timeline-Followback Interview^47^	Interview using memory prompts to determine substance use for the last 30 days.	Alcohol Use	15 min	30

Testing battery used for assessing drug use history for individual subjects. Individual test names are listed along with a description of the test, the variables tested for, and the duration of the test.

Participants' self-reported substance use was further corroborated with an additional measure of substance use (Timeline Follow Back [TLFB]^47^). Subjects were also given the Hollingshead Four-Factor Index of Socioeconomic Status^48^ to assess the influence of socioeconomic status (SES). Additionally, subjects were given the Mini-Mental State Examination to assess cognitive state,^49^ the Hamilton-D rating scale to assess depression,^50^ and the Wechsler Test of Adult Reading (WTAR) to estimate premorbid intellectual functioning.^51^ Based on the high co-occurrence of CB use with cigarette smoking and alcohol use, smoking and light alcohol use (<14 drinks/week for men, <7 drinks/week for women) were allowed.

Results from the Marijuana Smoking History Questionnaire (MSHQ)^52^ were used to create variables of interest related to CB use, including “Age of Onset” and “Lifetime Marijuana Use” (Average use/week×Number of years of use). We investigated the relationship between these variables in CB+ and CB− subjects and MTL subregional thickness. As frequency may be different over time (i.e., 5×/year for 2 years, then 1×/year for 25 years), the Lifetime Marijuana Use composite score was chosen as the most accurate representation of usage over many years.

We divided neuropsychiatric test scores into the following domains of cognitive function: *Memory Encoding, Delayed Memory, Processing Speed, and Executive Functioning* (see Fig. 4 for individual tests in each domain). Studies using these domains have been reported elsewhere.^37,53–55^ Raw scores in each test were first converted to Z scores [Z=(raw score-mean)/standard deviation] and then binned together to create a domain Z score by averaging the Z scores belonging to the cognitive tests in each domain.

**FIG. 1.**
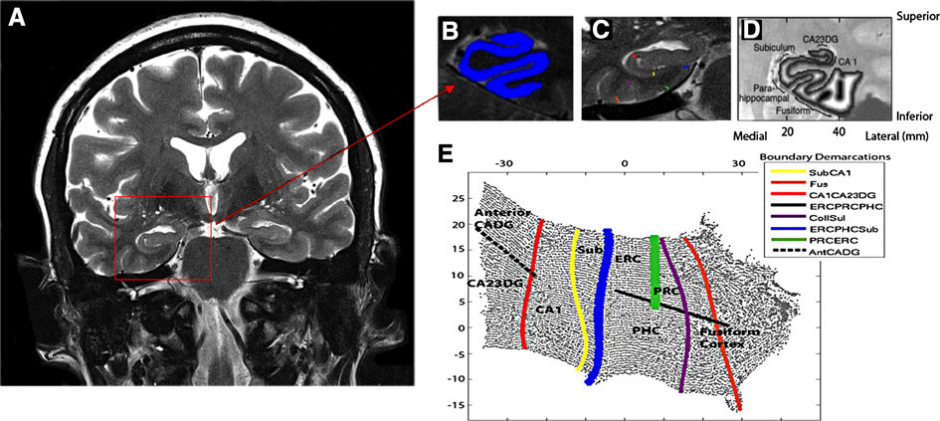
High-resolution hippocampal image processing and thickness calculations. **(A)** The goal of high-resolution hippocampal image processing is to isolate the strip of gray matter in the MTL that encompasses the subregions of the hippocampus proper and surrounding neocortex, shown in **(B)** in blue. This is done by manually segmenting cerebrospinal fluid and white matter and growing sequential layers of gray matter from the edge of white matter until the layer reach the cerebrospinal fluid boundary. **(C)** The boundaries between MTL subregions are marked according to anatomical landmarks. Because of limits in resolution in CA fields 2, 3, and DG we treat these regions as a single entity (CA23DG). Demarcations shown here include CA fields 2, 3, and the dentate gyrus (CA23DG) | CA1 (red), CA1 | subiculum (Sub; yellow), PHC | Sub (blue), fusiform gyrus (Fus; orange), Collateral sulcus (CollSul; purple) and PRC (green). **(D)** Boundary delineations were projected to their corresponding coordinates in flat map space. Each subregion is considered separately for cortical thickness calculations. Cortical thickness is visualized in in-plane space as a grayscale map of thickness values between maximum (white) and minimum (black) values. **(E)** To extract thickness, the distance from each voxel in in-plane space to the nearest nongray matter voxel was calculated. Next, the maximum distance value of two-dimensional voxels for the corresponding three-dimensional voxels across all layers was taken and multiplied by two to calculate mean thickness. Demarcations were projected from in-plane space to the corresponding location in 2D flat map space and then extended to form complete and smooth boundaries between subregions. CA1, cornu ammonis 1; ERC, entorhinal cortex; PHC, parahippocampal cortex; PRC, perirhinal cortex; MTL, medial temporal lobe.

All participants underwent a 45-min scan at the 3T Siemens Trio scanner located in the Center for Cognitive Neuroscience at the Semel Neuropsychiatric Institute using a 12-channel parallel coil. After scout and localizer scans were acquired (2 min), high-resolution fast spin echo (FSE) scans of the HC were acquired in an oblique coronal plane perpendicular to the long axis of the HC to ensure complete coverage (see Fig. 1a; FSE, repetition time [TR]=4800 ms, echo time [TE]=106 ms, field of view [FOV] 150, 512×512, NEX4, 26 slices, 2 mm thick, 0 mm spacing, 400×400 μm in-plane resolution). This sequence optimizes in-plane resolution, where the subregional variability is largest, while producing minimal variability through plane when the slices are precisely perpendicular to the long axis. We also acquired a Magnetization-Prepared Rapid Acquisition Gradient Echo (MPRage) scan for excellent gray–white contrast (T1-weighted volumetric study: TR 2300; TE: 2.93; flip angle 8°; FOV 256×256; bandwidth 210 Hz/px; 1-mm isotropic voxels) for visual reference during segmentation and ROI analyses.

We used cortical unfolding to improve visualization of the convoluted MTL cortex by flattening the entire three-dimensional volume into a two-dimensional flat map.^34,35,56^ Methods for high-resolution hippocampal analysis with unfolding are described in detail elsewhere.^35^ Boundary demarcations divided the following subregions encompassed by gray matter: cornu ammonis (CA) fields 1, 2, and 3, the DG, subiculum (Sub), ERC, perirhinal cortex, parahippocampal cortex, and the fusiform gyrus (Fus) (Fig. 1b–e). This strip of gray matter is used as the input for the unfolding procedure, an iterative algorithm based on multidimensional scaling (http://ccn.ucla.edu/wiki/index.php/Unfolding). Cortical thickness within subregions was averaged over both hemispheres. The following formula was used to normalize hippocampal thickness values to intracranial volume (ICV) estimates: ICV-corrected thickness=[(thickness in mm/ICV in mm^3^)×10^6^]. Multiplying by 10^6^ results in values at the same order of magnitude as original thickness estimates.

We used FreeSurfer 6.0 to process T1 MRI scans (http://surfer.nmr.mgh.harvard.edu). Based on the statistical maps from the data set, The Open Access Series of Imaging Studies (OASIS; https://www.oasis-brains.org), we created an ROI of parietal cortex. For volumetric calculations of volume in inferior parietal lobe, we used FreeSurfer on whole brain T1-weighted scans. After the automated portion of the FreeSurfer pipeline was complete, each participant's scan was visually checked for accuracy. Minimal manual edits were completed by a single individual when necessary. ICV values from FreeSurfer were used to normalize volume in the parietal lobe volumes. Because FreeSurfer parcellation results in separate volumes for superior and inferior parietal lobe volumes, results from the two ROIs were used to create an average parietal lobe ROI for each subject.

### Statistical analyses

Demographic variables between groups were compared using *t*-tests for continuous measures and chi-squared tests for categorical measures. General linear models were used to test for significant group differences in thickness for each of the MTL subregions, with sex, age, WTAR, cigarette use, and education included as covariates. Differences in neuropsychological performance in each domain were also tested with general linear models, with the same covariates shown above. A similar model examined differences in the volumes of parietal lobes between CB+ and CB− groups, controlling for the above covariates as well as total ICV. In addition to the standard statistics, we also present effect sizes (ES; Cohen's d) for all group differences. Statistical significance was set at *p*=0.05 for all models.

## Results

Clinical and demographic characteristics are described in Table 2. Results from the MSHQ (Table 1) indicated an average age of onset of 17.7±4.2 years of age and lifetime cannabis use of 11.3±13.0 years duration. Groups did not differ in age, gender, or MMSE scores. However, the groups differed in number of years of education, cigarette usage, and WTAR performance; these variables were included as covariates in general linear models examining differences in the mean between the two groups.

**Table 2.** **Demographic and Clinical Characteristics of Study Participants (mean/n±SD)**

	CB+	CB−	Between-group statistics
Cohort characteristics	*n*=24	*n*=26	*t*(48)/χ^2^(1), *p*-value
Mini-Mental State Examination score	28.0±1.6	28.4±1.7	0.82, 0.42
Age, years	65.4±7.2	67.7±7.1	1.12, 0.27
Educational achievement, years	15.3±2.0	16.6±2.3	2.04, 0.05^**^
Female sex, *n* (%)	8 (33)	12 (46)	1.92, 0.17
Cigarette smoker, *n* (%)	14 (58)	7 (27)	5.05, 0.02^**^
Hamilton Depression Scale score	7.0±7.5	4.9±5.3	1.16, 0.25
WTAR	35.7±9.6	41.2±7.4	2.24, 0.03^**^
Hollingshead coding	5.4±1.7	5.8±1.7	0.92, 0.36
Cannabis use		—	—
Onset of regular use (age in years±SD)	17.7±4.2	—	—
Duration of use (years±SD)	11.3±13.0	—	—
Lifetime use (days±SD)	4181.2±4784.6	—	—
Length of abstinence (years±SD)	29.9±6.0	—	—
Lifetime alcohol use (no. of standard drinks/month±SD)	21.6±12.0	19.8±11.3	−0.54, 0.59

^**^ *p*<0.05.

SD, standard deviation; WTAR, Wechsler Test of Adult Reading.

**FIG. 2.**
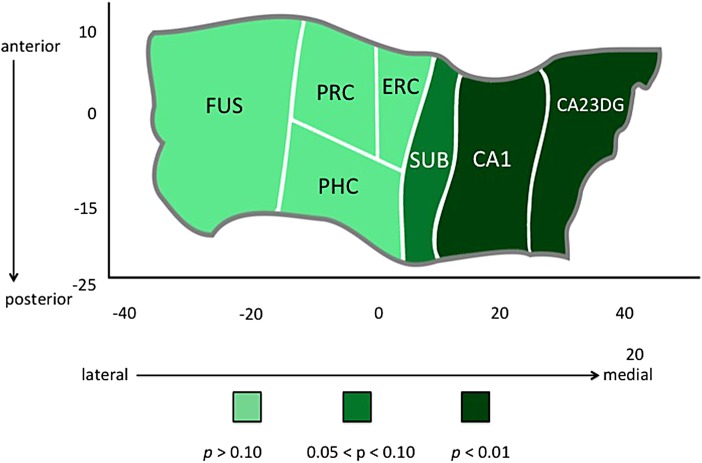
Hippocampal complex unfolding reveals relationship between CB use and subregional hippocampal morphology in late life. A cortical unfolding procedure was used to produce a flat map of the hippocampal complex. Regions are color coded according to the strength of the statistical association between CB group and cortical thickness in individual subregions within the hippocampus and surrounding neocortex. CB, cannabis.

**FIG. 3.**
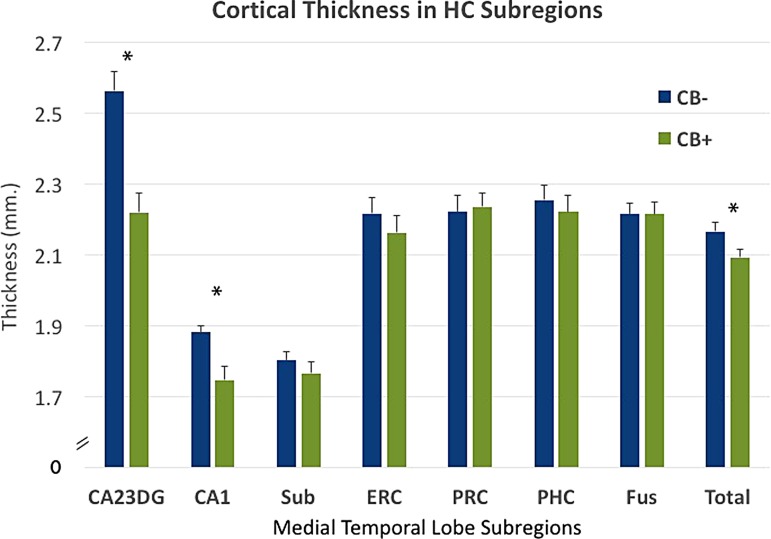
Subregional cortical thickness in individual subregions for CB+ and CB− groups. CB+ subjects showed thinner cortex in subfields CA1 [F(1,42)=9.96, *p*=0.003; ES=0.98] and CA23DG [F(1,42)=23.17, *p*<0.0001; ES=1.49] and thinner hippocampus averaged over all subregions [F(1,42)=8.49, *p*=0.006; ES=0.90]. Group averages in subregional thickness are displayed with standard error bars. *p<0.05. ES, effect size.

CB+ subjects had thinner cortex in subfields CA1 [F(1,42)=9.96, *p*=0.003; ES=0.98] and CA23DG [F(1,42)=23.17, *p*<0.0001; ES=1.49] (Fig. 2) and thinner HC averaged over all subregions [F(1,42)=8.49, *p*=0.006; ES=0.90] (Fig. 3). WTAR scores were significantly lower in CB+ subjects [*t*(47)=2.24, *p*=0.03], but even after removing the most extreme values in WTAR (to better match groups on WTAR performance and ensure that this factor was not driving morphological differences between groups), CA1 and CA23DG thickness were still significantly lower in CB+ subjects [CA1: F(1,35)=9.30, *p*=0.004, S=1.03; CA23DG: F(1,35)=23.95, *p*<0.0001, ES=1.65]. The two groups did not differ in SES either in early childhood or adulthood.

**FIG. 4.**
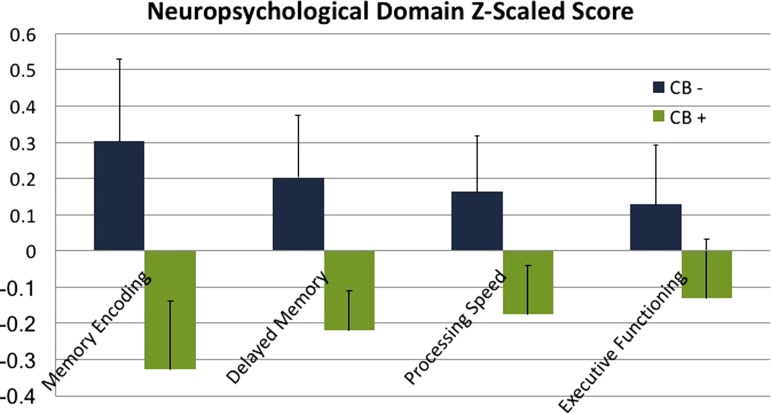
Neuropsychological domain scores, Z-scaled for CB+ and CB− groups. Domains were created using the following tests within each domain: *Memory Encoding* (Buschke-Fuld selective reminding test, consistent long-term retrieval; Wechsler Memory Scale-II, Logical Memory I and Verbal Paired Associates I), *Delayed Memory* (Wechsler Memory Scale: Logical Memory II and Verbal Paired Associates II; Rey–Osterrieth Complex Figure, Delayed Recall; Buschke-Fuld Selective Reminding Test, Delayed Recall), *Processing Speed* (Trailmaking Test, Part A; Stroop Test, Word Reading Speed; Wechsler Adult Intelligence Scale-III Digit Symbol), and *Executive Functioning* (Trailmaking Test Part B; Verbal Fluency FAS and Animal Naming tests; Stroop Test, Interference). Group averages and standard errors are displayed for all neuropsychological test domains. Differences between the groups were not significant, however, CB+ group averages were less than CB− averages across all domains.

Additionally, although “Lifetime Alcohol Use” was not significantly different between the two groups [*t*(48)=0.54, *p*=0.6], group models were run an additional time with this variable added as an covariate to test whether differences in alcohol use patterns were driving group differences. However, the pattern of significant differences in subregional cortical thickness between groups remained unchanged [CA23DG [F(1,41)=25.32, *p*<0.0001; ES=1.37 and CA1 [F(1,41)=9.67, *p*=0.003; ES=1.05]. In contrast, there were no group differences in the control parietal lobe region between the two groups [F(1,41)=0.45, *p*=0.51; ES=0.21]. No relationship between age of onset of cannabis use or lifetime cannabis use was found with thickness metrics. Differences between groups were not statistically significant across any neuropsychological measures [Memory Encoding Domain: F(1,42)=0.90, *p*=0.35, ES=0.29; Delayed Memory Domain: F(1,42)=0.94, *p*=0.34, ES=0.30; Processing Speed Domain: F(1,42)=1.33, *p*=0.26, ES=0.36; Executive Function Domain: F(1,42)=0.05, *p*=0.82, ES=0.07]. No neuropsychological domains showed a significant relationship with cortical thickness in any subregions. However, CB+ group averages were lower than CB− group averages across all neuropsychological measures (Fig. 4).

## Discussion

These results provide the first evidence of an association between early life CB use and later morphology changes in the medical temporal lobe, several decades after cessation of usage. Participants who reported heavy CB use in early life showed thinner cortex in hippocampal subregions CA1, 2, 3, and the DG. Morphological differences between groups were regionally specific to CA and DG regions and were not present in neocortical regions of the MTL, including entorhinal, perirhinal, and parahippocampal cortices. Additionally, cortical thickness in parietal cortex, a region relatively low in CB1 receptors but still subject to age-related changes, showed no differences between groups. These results underscore the persistent nature of adolescent-induced brain changes due to heavy CB use, and the rising need to understand how these changes interact with brain aging.

Our findings of thinner hippocampal cortex restricted to CA1, 2, 3 and the DG are relevant in relation to the functional impairment shown in chronic CB users during adolescence. Hippocampal subregions follow synaptic projections from the ERC through the DG, CA3, CA2, and then to Sub with each synaptic connection representing a specific function in the information processing pathway.^57,58^ Hippocampal subregions, CA1, CA3, and the DG, support the representation and encoding of novel information, allowing the HC to organize information and support short-term memory processes.^59^

In adolescent and early life CB users, memory impairment is one of the most frequent problems observed with persistent CB use,^21^ and it may be linked to specific structural alterations in CA3 and the DG. Although no differences in neuropsychiatric domain scores were found in the present study, lower scores in CB users were noted for every domain (Fig. 4); a larger sample size in future studies will help elucidate this relationship more fully.

Neuroimaging investigations on the structural effects of CB use on the brain have been inconsistent.^32^ Changes in gray or white matter density have been reported in different locations in frontal and parietal lobes without overlapping results across studies.^60–62^ These discrepancies might be due to heterogeneity in sample characteristics, individual differences in comorbid substance use, amount of consumption, or methodological differences in data processing.^17,63^

Changes in the HC/parahippocampal complex have often been reported^33,62,64,65^ and highlights the rationale behind the present investigation on the MTL, which was intended to investigate the relationship between early life CB use and late-life brain morphology. Although the precise mechanisms underlying the effects of CB on the HC are not fully understood, animal studies have shown that THC accumulates in neurons,^66^ with long-term exposure to THC resulting in neurotoxic changes in hippocampal microstructure,^67^ and there is particular concern regarding potential age-related interaction in the MTL, a brain area with high susceptibility to age-related morphology changes in late life.^68^ Adding to this concern, the fact that subjects in the present study had been rare or abstinent users for several decades suggests that CB-induced morphological changes from heavy adolescent exposure may have very long-term negative consequences.

Retrospective assessment of any drug use with no corroboration is challenging considering the life-long experiences and exposures among individual subjects. It is also noted that comparing parietal cortex, which is larger in size than the HC, is not perfectly matched to the sensitivity of examining hippocampal subregions. Selection bias is another confound we recognize; one cannot know for certain if the differences in hippocampal atrophy between groups are due to pre-existing differences before CB use onset.

However, since the results held true after regression of the variables most likely to reflect baseline differences between groups (WTAR scores, gender, and cigarette use) as well as the specificity of results to CB1-rich brain regions, the findings presented in this study are not easily attributed to pre-existing group differences. Future studies should attempt to reproduce our CB-dependent effects in larger samples. Currently, the Adolescent Brain Cognitive Development study (https://abcdstudy.org) is prospectively following brain development with the largest long-term study of child brain development in the United States.

With increased usage, potency, and research indicating CB affects the HC,^31^ investigating the long-term effects of adolescent use in an aging population is essential for understanding the long-term consequences of heavy, early life CB exposure. Approximately 9% of persons who experiment with CB will become addicted.^69^ Our results provide evidence of neuroanatomical alterations in the hippocampi of ex-CB users and underscore the importance of assessing subregional hippocampal morphology. They also underscore the persistent nature of adolescent-induced brain changes due to CB use, and the rising need to understand how these changes interact with brain aging. Future studies should examine the effects of these morphological differences longitudinally to uncover the functional and behavioral implications of these brain differences.

## Abbreviations Used

BP: blood pressure
CA: cornu ammonis
CB: cannabis
CB1: cannabinoid 1
DG: dentate gyrus
ERC: entorhinal cortex
ES: effect size
FSE: fast spin echo
HC: hippocampus
ICV: intracranial volume
MRI: magnetic resonance imaging
MSHQ: Marijuana Smoking History Questionnaire
MTL: medial temporal lobe
PHC: parahippocampal cortex
ROI: region of interest
THC: Δ9-tetrahydrocannabinol
